# DO OLDER PATIENTS “WARN” THAT THEY WILL SUFFER A NEW FRACTURE?

**DOI:** 10.1590/1413-785220233105e266844

**Published:** 2023-12-18

**Authors:** SAMUEL BICHARA MELIN, MARCELA RODRIGUES SANTOS DO NASCIMENTO, ALFREDO DOS SANTOS, CAIO ZAMBONI

**Affiliations:** 1 Santa Casa de Misericordia de São Paulo, Department of Orthopedics and Traumatology, São Paulo, SP, Brazil; 2 Santa Casa de Misericordia de São Paulo, School of Medical Sciences, São Paulo, SP, Brazil; 3 Santa Casa de Misericordia de São Paulo, Department of Orthopedics and Traumatology, Trauma Surgery Group, São Paulo, SP, Brazil

**Keywords:** Osteoporotic Fracture, Osteoporosis, Aged, Secondary Prevention, Fraturas por Osteoporose, Osteoporose, Idoso, Prevenção Secundária

## Abstract

**Objective:**

To evaluate whether patients older than 60 years admitted for fracture treatment had a history of previous fracture, a diagnosis of osteoporosis, or were under treatment for bone fragility.

**Methods:**

Retrospective study including 100 patients older than 60 years with fracture. Fracture location, bone densitometry within the past two years, previous diagnosis and osteoporosis treatment, and previous fracture within the past five years were assessed. Using Fisher’s test, it was evaluated whether there was an association between previous fracture and osteoporosis treatment.

**Results:**

The most prevalent fracture was in the proximal femur (48%). Of the patients, 18% had fracture in the last five years, with 22% of them diagnosed with osteoporosis, and 22% under treatment. Previous fracture in the last five years was not associated with having a diagnosis of osteoporosis, having had bone densitometry, or being under treatment for osteoporosis.

**Conclusion:**

Among patients with previous fracture, only 22% were aware of their diagnosis of osteoporosis, and less than 25% of them were under bone fragility treatment. Previous fracture in the past five years had no association with having a diagnosis of osteoporosis, having had bone densitometry, or being on osteoporosis treatment. ***Level of Evidence III, Retrospective Study.***

## RESUMO

**Objetivo:**

Avaliar se os pacientes com mais de 60 anos internados para tratamento de fraturas têm história de fratura prévia, diagnóstico de osteoporose ou se estão em tratamento para fragilidade óssea.

**Métodos:**

Estudo retrospectivo que inclui 100 pacientes maiores de 60 anos com fratura. Avaliamos a localização da fratura, a densitometria óssea nos últimos dois anos, o diagnóstico e os tratamentos anteriores de osteoporose, assim como a presença de fratura prévia nos últimos cinco anos. Através de testes de Fisher avaliamos se houve associação entre fratura prévia e tratamento de osteoporose.

**Resultados:**

A fratura mais prevalente foi na extremidade proximal do fêmur (48%). Do total de pacientes, 18% tiveram fratura nos últimos cinco anos, sendo que 22% deles tinham diagnóstico de osteoporose e 22% estavam em tratamento. Fratura prévia nos últimos cinco anos não teve associação com diagnóstico de osteoporose, realização de densitometria óssea ou tratamento para osteoporose.

**Conclusão:**

Entre os pacientes com fratura prévia, apenas 22% estavam cientes do diagnóstico de osteoporose, e menos de 25% deles estavam em tratamento para fragilidade óssea. Não houve associação de fratura prévia nos últimos cinco anos com diagnóstico de osteoporose, realização de densitometria óssea ou tratamento para osteoporose. **Nível de Evidência III, Estudo Retrospectivo.**

## INTRODUCTION

Population’s aging is a worldwide phenomenon. This trend leads to a greater concern with diseases related to this age group, including osteoporosis,^1^ a disease characterized by decreased bone mass and deterioration of the microarchitecture of bone tissue, with a consequent increase in fragility. ^(^ ^2^ ^),(^ ^3^

Osteoporotic fractures produce serious physical and psychological consequences, affect the quality of life of patients with osteoporosis and their caregivers, and have a high socioeconomic impact. Among these, proximal femur fractures bring with them high morbidity and mortality. ^(^ ^3^ ^)-(^ ^5^ When individuals suffer their first fracture caused by fragility, they are diagnosed with “established osteoporosis.” From this moment it is known that the risk of a new fracture increases considerably compared with patients without previous fracture, emphasizing the importance of osteoporosis treatment in these patients. ^(^ ^2^ ^),(^ ^5^ ^), (^ ^6^ ^), (^ ^7^ ^), (^ ^8^ ^), (^ ^9^ ^), (^ ^10^ However, the lack of diagnostic accuracy and guidance of appropriate osteoporosis treatment remain high, even in patients who have already had a first osteoporotic fracture. ^(^ ^5^ ^),(^ ^7^ ^),(^ ^10^ ^), (^ ^11^ ^), (^ ^12^ ^), (^ ^13^

### Objectives

Our primary objective was to evaluate whether patients aged over 60 years hospitalized for surgical treatment of fractures had a history of previous fracture in the last five years, and if so, whether they had a diagnosis of osteoporosis or were undergoing treatment to reduce bone fragility. Our secondary objectives were to perform an epidemiological characterization of these patients. In addition, we evaluated whether patients with a history of previous fracture had a greater association with awareness of osteoporosis diagnosis, if they had undergone bone densitometry in the last two years, and were undergoing some type of specific treatment, we compared these data with patients who did not suffer previous fractures.

## METHODS

This is a retrospective cohort study, in which, after approval by the Ethics Committee on Research in Human Beings of the Irmandade da Santa Casa de Misericórdia de Sao Paulo (CAAE: 65619717.1.0000.5479), we evaluated the cases of patients aged over 60 years and diagnosed with fracture, hospitalized for surgical treatment at the Department of Orthopedics and Traumatology of Santa Casa de Misericórdia de São Paulo from January 2021 to December 2021.

After excluding cases of fracture in cancer patients and patients who were unable to answer the questionnaire or who refused to sign an informed consent form, 100 patients were included in the study. The questionnaire applied to the patients included the following information: age, sex, location of the fracture, mechanism of the trauma, place of the fall that resulted in the fracture, performance of bone densitometry in the last two years, previous diagnosis of osteoporosis, use of medications for treating osteoporosis (vitamin D, calcium, and bisphosphonates), previous fracture in the last five years, and the location of such fracture.

The evaluated characteristics were described with absolute and relative frequencies for all older adults evaluated. We also evaluated, using Fisher’s exact tests, ^(^ ^14^ whether a previous fracture was more associated or not with being aware of the diagnosis of osteoporosis, whether bone densitometry was performed in the last two years and whether the patient is undergoing some type of supplementation with calcium, vitamin D, or bisphosphonate treatment.

The analyses were performed with the IBM-SPSS software for Windows version 22.0 and tabulated with the Microsoft-Excel 2010 software, and the tests were performed with a 95% significance level.

## RESULTS

Table 1 shows the detailed description of the data of the 100 patients evaluated. The most prevalent age group was aged from 71 to 80 years (39%). Most participants were females (65%), and the most prevalent fracture was that of the proximal femur (48%), followed by fractures in the distal radius (13%) and proximal humerus (12%). The most frequent trauma mechanism was falling at ground level at home (54%), and the bedroom and bathroom were the rooms with the highest number of accidents (31.5% and 27.9%, respectively). Among the 100 patients (100), only 13% underwent bone densitometry in the last two years, and less than 20% of the patients were being treated with calcium, vitamin D, or bisphosphonates. Only 16% of the patients in our series had a diagnosis of osteoporosis, 16% were under calcium supplementation and 12% were under vitamin D supplementation.

**Table 1:** Description of the characteristics evaluated for all patients.

Characteristic	Description (N = 100)
**Age (years)**	
61 to 70 years	33 (33.0)
71 to 80 years	39 (39.0)
> 80 years	28 (28.0)
**Sex**	
Female	65 (65.0)
Male	35 (35.0)
**Fracture**	
Clavicle	1 (1.0)
Spine	1 (1.0)
Elbow	6 (6.0)
Distal femur	1 (1.0)
Proximal femur	48 (48.0)
Leg bones	1 (1.0)
Patella	1 (1.0)
Distal tibia	1 (1.0)
Distal radius	13 (13.0)
Sacrum	2 (2.0)
Proximal tibia	2 (2.0)
Ankle	11 (11.0)
Proximal humerus	12 (12.0)
**Trauma mechanism**	
Fall at ground level at home	54 (54.0)
Fall at ground level on the street	32 (32.0)
Run over by a vehicle	9 (9.0)
Fall of the ladder	5 (5.0)
If a fall at home, which room?*	
Living room	12 (22.2)
Bedroom	17 (31.5)
Bathroom	14 (25.9)
Kitchen	7 (13)
Backyard	4 (7.4)
**Bone densitometry in the last two years?**	
No	87 (87.0)
Yes	13 (13.0)
**Previous diagnosis of osteoporosis?**	
No	84 (84.0)
Yes	16 (16.0)
**Calcium supplementation**	
No	84 (84.0)
Yes	16 (16.0)
**Vitamin D supplementation**	
No	88 (88.0)
Yes	12 (12.0)
**Bisphosphonate treatment**	
No	95 (95.0)
Yes	5 (5.0)
**Any fractures in the last five years?**	
No	82 (82.0)
Yes	18 (18.0)
Which previous fracture?*	
Distal radius	4 (22.2)
Proximal humerus	2 (11.1)
Proximal humerus	7 (38.9)
Other	5 (27.8)

Data expressed as n (%); * Only for valid cases.

Among the 100 patients, 18 had previous fractures in the last five years, and the most common were proximal femur (7) and distal radius (5). Among these patients, only 22% had a previous diagnosis of osteoporosis, and less than 25% of them were under calcium (16.7%) or vitamin D (22.2%) supplementation, and none were under bisphosphonate treatment (Table 2).

**Table 2:** Description of the cases that had a previous fracture, without statistical association with awareness of the diagnosis of osteoporosis, having undergone bone densitometry in the last two years, and undergoing some type of treatment with calcium, vitamin D, or bisphosphonate.

Parameter	Any fractures in the last five years?		p
	No (N = 82)	Yes (N = 18)	
**Bone densitometry in the last two years?**			0.699
No	72 (87.8)	15 (83.3)	
Yes	10 (12.2)	3 (16.7)	
**Previous diagnosis of osteoporosis?**			0.480
No	70 (85.4)	14 (77.8)	
Yes	12 (14.6)	4 (22.2)	
**Calcium supplementation**			> 0.999
No	69 (84.1)	15 (83.3)	
Yes	13 (15.9)	3 (16.7)	
**Vitamin D supplementation**			0.221
No	74 (90.2)	14 (77.8)	
Yes	8 (9.8)	4 (22.2)	
**Bisphosphonate treatment**			0.582
No	77 (93.9)	18 (100)	
Yes	5 (6.1)	0 (0)	

Data expressed as n (%); Fisher’s exact test

Previous fracture in the last five years had no statistically significant association with awareness of the diagnosis of osteoporosis, having undergone bone densitometry in the last two years, and undergoing some type of treatment with calcium, vitamin D, or bisphosphonate (p < 0.05).

## DISCUSSION

The fractures most commonly associated with bone fragility are fractures at the proximal femur, vertebral body, proximal humerus, and distal radius. ^(^ ^9^ The most prevalent fracture among our patients was the fracture in the proximal femur and the most frequent trauma mechanism was the fall at ground level at home, with the bedroom and bathroom as the rooms with the highest number of falls. The literature series corroborate our findings that the most common trauma mechanism of these fractures is the fall at ground level at home. ^(^ ^1^ ^),(^ ^3^ ^),(^ ^4^ ^),(^ ^15^ Note that the number of fractures of the distal radius was lower than expected, but we noticed that many patients were excluded from the study for being under 60 years of age.

We followed the cases of patients aged over 60 years hospitalized for surgical treatment of fractures in our service for one year. During this period, we had 48 cases of fracture in the proximal femur, a lower number than that presented in other series in the literature that followed similar cases for the same period in the same service. ^(^ ^1^ ^),(^ ^4^ One of the possible explanations for this is the social isolation caused by the COVID-19 pandemic. Silva et al. ^(^ ^16^ showed a significant reduction in the incidence of hip fractures in individuals aged over 60 years in Brazil during the social isolation due to COVID-19.

Only 13% of our patients underwent bone densitometry in the last two years. This data draws our attention, since the decrease in bone mass is happening silently and therefore an active search should be made evaluating patients over 60 years, especially females. ^(^ ^3^ The low rate of diagnosis and specific treatment for osteoporosis evinced leads us to a serious problem. Vitamin D plays an important role in calcium metabolism and, consequently, in bone mineralization and osteoporotic conditions. Its deficiency is, therefore, an important risk factor for fractures in older adults. Its use has been recommended as a way to prevent fractures in older adults with osteoporosis. ^(^ ^15^ However, this is not always routinely performed in public health, as our findings confirm. Guerra et al. ^(^ ^15^ showed that patients with fractures at the proximal femur had significantly reduced serum vitamin D levels compared with patients without fractures of the proximal femur.

Patients aged over 60 years with any fracture have a 50% to 100% higher risk of having another fracture in the future, and the occurrence of fracture at the proximal femur increases the risk of subsequent fracture by six times. ^(^ ^5^ In our series, 18% of the patients had a previous fracture in the last five years, and 38% of them were in the proximal femur. Only 22% of them were aware of the diagnosis of osteoporosis, and less than 25% of them were on calcium or vitamin D supplementation, with none of them taking bisphosphonate. Previous fracture in the last five years was not associated with awareness of the diagnosis of osteoporosis, having undergone bone densitometry in the last two years, or undergoing some type of drug treatment or specific supplementation.

These findings are worrisome and demonstrate that the treatment of osteoporosis in patients after fracture, despite the numerous publications warning about the subject in the literature, remains less than ideal. ^(^ ^5^ ^),(^ ^7^ ^),(^ ^10^ ^),(^ ^12^ ^),(^ ^13^ ^),(^ ^17^

A possible explanation for this fact is the low participation of orthopedists in the treatment of osteoporosis. Although orthopedists identify osteoporosis after a fracture, the disease is often treated by other physicians such as generalists, gynecologists, rheumatologists, endocrinologists, and geriatricians. The lack of involvement of orthopedists can be attributed to their reluctance to take responsibility for a chronic disease, their focus on treating the consequences of the disease rather than its causes, or, more possibly, a lack of knowledge about treatment. Zamboni et al. ^(^ ^13^ showed that less than half of orthopedists and traumatologists in Brazil make the diagnosis and secondary prevention for osteoporotic fractures, only 0.8% treat these patients correctly, and 47% refer them to clinical specialties. Studies show that the involvement of orthopedic surgeons improves osteoporosis treatment rates. ^(^ ^7^ We believe that the results of this study, which are in line with the literature, show a continuous loss of health promotion opportunity, failing to prevent new fractures in older patients, a situation that, unfortunately, remains common in our health system.

A possible solution is to draw the attention of orthopedic surgeons about the importance and relevance of the topic, and reinforce that they should not only treat the fractures of these patients, but also start the treatment of osteoporosis to prevent new ones.

## CONCLUSION

In this study, 18% of patients had a previous fracture in the past five years, with only 22% of them being aware of the diagnosis of osteoporosis, and less than 25% of them were being treated with calcium or vitamin D, with none taking bisphosphonate. The most prevalent fracture was the proximal femur fracture and the most frequent trauma mechanism was the fall at ground level at home, with the bedroom and bathroom as the rooms with the highest number of accidents. Only 13% of patients underwent bone densitometry in the last two years, and less than 20% were being treated with calcium, vitamin D, or bisphosphonates. Previous fracture in the last five years was not associated with awareness of the diagnosis of osteoporosis, having undergone bone densitometry in the last two years, or undergoing some type of treatment for bone fragility with calcium, vitamin D, or bisphosphonate.

## References

[1] Daniachi D, S A, Ono NK, Guimarães RP, Polesello GC, Honda EK (2015). Epidemiology of fractures of the proximal third of the femur in elderly patients. Rev Bras Ortop.

[2] Petrella RJ, Jones TJ (2006). Do patients receive recommended treatment of osteoporosis following hip fracture in primary care. BMC Fam Pract.

[3] Riera R, Trevisani VFM, Ribeiro JPN (2003). Osteoporosis - the importance of preventing falls. Rev Bras Reumatol.

[4] Hungria JS, Dias CR, Almeida JDB (2011). Epidemiological characteristics and causes of proximal femoral fractures among the elderly. Rev Bras Ortop.

[5] Fortes EM, Raffaelli MP, Bracco OL, Takata ETT, Reis FB, Santili C, Lazaretti-Castro M (2008). High morbid-mortability and reduced level of osteoporosis diagnosis among elderly people who had hip fractures in São Paulo City. Arq Bras Endocrinol Metabol.

[6] Bahl S, Coates PS, Greenspan SL (2003). The management of osteoporosis following hip fracture have we improved our care?. Osteoporos Int.

[7] Talbot JC, Elener C, Praveen P, Shaw DL (2007). Secondary prevention of osteoporosis calcium, vitamin D and bisphosphonate prescribing following distal radial fracture. Injury.

[8] Nojiri S, Burge RT, Flynn JA, Foster SA, Sowa H (2013). Osteoporosis and treatments in Japan management for preventing subsequent fractures. J Bone Miner Metab.

[9] Bawa HS, Weick J, Dirschl DR (2015). Anti-osteoporotic therapy after fragility fracture lowers rate of subsequent fracture analysis of a large population sample. J Bone Joint Surg Am.

[10] Viprey M, Caillet P, Canat G, Jaglal S, Haesebaert J, Chapurlat R, Schott AM (2015). Low osteoporosis treatment initiation rate in women after distal forearm or proximal humerus fracture a healthcare database nested cohort study. PLoS One.

[11] Woo SH, Park KS, Choi IS, Ahn YS, Jeong DM, Yoon TR (2020). Sequential bilateral hip fractures in elderly patients. Hip Pelvis.

[12] Khan AA, AbuAlrob H, Tariq F, Tauqir M, Zalzal P, M'Hiri I (2021). Osteoporosis treatment rate following hip fracture in a community hospital. Arch Osteoporos.

[13] Zamboni C, Carvalho MS, Pires EA, Durigan JR, Fucs PMMB, Mercadante MT (2018). Are traumatologists treating osteoporosis to prevent new fractures in Brazil. Acta Ortop Bras.

[14] Kirkwood BR, Sterne JAC (2006). Essential medical statistics.

[15] Guerra MTE, Feron ET, Viana RD, Maboni J, Pastore SI, Castro CC (2016). Elderly with proximal hip fracture present significantly lower levels of 25-hydroxyvitamin D. Rev Bras Ortop.

[16] Silva AC, Santos GS, Maluf EMCP, Borba VZC (2022). Incidence of hip fractures during the COVID-19 pandemic in the Brazilian public health care system. Arch Osteoporos.

[17] Rinat B, Rubin G, Orbach H, Giwnewer U, Rozen N (2016). Can orthopedic surgeons help create a better head start for osteoporosis treatment after hip fracture. Medicine (Baltimore).

